# Characteristics and outcomes of children with dissociative (conversion) disorders in western China: a retrospective study

**DOI:** 10.1186/s12888-021-03045-0

**Published:** 2021-01-12

**Authors:** Zhixu Fang, Yuhang Li, Lingling Xie, Min Cheng, Jiannan Ma, Tingsong Li, Xiujuan Li, Li Jiang

**Affiliations:** 1 Department of Neurology, Children’s Hospital of Chongqing Medical University, No. 136, Zhongshan Er Road, Yuzhong District, Chongqing City, 400014 China; 2 Ministry of Education Key Laboratory of Child Development and Disorders, Chongqing, 400014 China; 3 National Clinical Research Center for Child Health and Disorders, Chongqing, 400014 China; 4 China International Science and Technology Cooperation Base of Child Development and Critical Disorders, Chongqing, China; 5 Chongqing Key Laboratory of Pediatrics, Chongqing, China

**Keywords:** Dissociative (conversion) disorders, Children, Psychiatric, Socio-cultural environmental factors, Outcomes

## Abstract

**Background:**

Dissociative (conversion) disorder in children is a complex biopsychosocial disorder with high rates of medical and psychiatric comorbidities. We sought to identify the characteristics and outcomes of children with dissociative (conversion) disorders in western China.

**Methods:**

We conducted a retrospective cohort study of 66 children admitted with dissociative (conversion) disorders from January 2017 to July 2019, and analyzed their clinical characteristics, socio-cultural environmental variables, and personality and psychiatric/psychological characteristics. Binary logistic regression was used to analyze the variables associated with clinical efficacy.

**Results:**

Of these 66 patients, 38 (57.6%) were male and 28 (42.4%) were female, 46 (69.7%) had an antecedent stressor, 30 (45.5%) were left-behind adolescents, and 16 (24.2%) were from single-parent families. In addition, 30 patients (45.5%) were not close to their parents, 38 patients (59.4%) had an introverted personality, and 34 (53.1%) had unstable emotions. Thirteen families (19.7%) were uncooperative with the treatment. Patients who had cormorbid anxiety or depression exhibited significantly lower cognitive ability (*P* < 0.01). Logistic regression found that better treatment outcomes were positively associated with having a close relationship with parents, parental cooperation with treatment, and having a father with a lower level of education (i.e., less than junior college or higher).

**Conclusions:**

The characteristics and outcomes of children with dissociative (conversion) disorders are related to socio-cultural environmental variables and psychiatric/psychological factors. Timely recognition and effective treatment of dissociative (conversion) disorders are important.

## Background

Dissociative (conversion) disorders, which were formerly known as hysteria, are one of the most common classes of psychiatric disorders in the world. The latest Diagnostic and Statistical Manual of Mental Disorders - Fifth edition (DSM-5) describes dissociative disorders as broadly involving impairments in the integration of all of the following: consciousness, memory, identity, emotion, perception, body representation, motor control, and behavior. The major diagnostic criteria for conversion disorders is one or more symptoms of altered voluntary motor or sensory function that cause distress or significant disruption of daily life, where the symptom or deficit is not better explained by another medical or mental disorder. Typical conversion symptoms include motor weakness, abnormal movements, non-epileptic seizures, and loss of sensory perception [1].

There has been a growing awareness of dissociative (conversion) disorders in children in recent decades. However, no solid data are currently available on the prevalence of dissociative (conversion) disorders in children and adolescents in China. Dissociative disorders are rare in children, but conversion disorders account for the majority of them, especially in developing countries [2]. Studies have shown that dissociative (conversion) disorders have a complex relationship with the patients’ body, mind, and socio-cultural environment [3], and that stressful life events, traumas, and adjustment difficulties have a positive association with subsequent dissociative and conversion symptoms in children [4, 5]. The most common stressors or traumas are family conflict, parental divorce, learning problems, refusing to attend school, bullying, scolding, and punishment [6, 7]. In addition, parents’ marital status, rearing style, family economic conditions, and a child being left behind by parents are also related to the manifestation of dissociative (conversion) disorders [3].

Due to the one-child policy, most children in China are only-children, and people are becoming more concerned that these children will gradually develop a self-centered, proud, and grumpy personality. Furthermore, the economic and cultural level of western China is lower than that of eastern China, and the problem of left-behind children is prominent. Migrant workers from rural areas have swarmed into economically prosperous cities to seek better work opportunities, and they have had to leave their children behind in their hometowns, having little communication with them. Some of these children have been found to have the personality characteristics of emotional instability and behavioral impulsivity [2]. Because they lack an adequate strategy to deal with stressors effectively, they commonly have coexisting mood impairments, especially anxiety, depressed mood, or irritability [3]. In addition, as in other parts of China, adverse childhood experiences (e.g., physical abuse, sexual abuse, neglect, and family violence) have a strong correlation with psychoform/somatoform dissociation in these patients, and dissociation is positively correlated with the number of types of trauma [8–10]. Theoretically, the development of dissociative (conversion) disorders might be due to unresolved psychological conflicts that are converted into physical or psychiatric symptoms, which have gained social acceptance and protect the child from conflict or painful memories [11]. Being ill also brings relief from sensations that are unpleasant and difficult to accept (primary benefits), reduce high expectations, and increase the attention of loved ones, e.g., parental indulgence (secondary benefits) [12]. Previous studies have found that children with dissociative (conversion) disorders often have other comorbid somatic symptoms and psychiatric disorders [13–16]. In addition, cognitive deficits may develop in some patients with dissociative (conversion) disorders [13].

Dissociative (conversion) disorders may result in significant social, economic, and health burdens on children. Therefore, understanding the clinical characteristics, socio-cultural and environmental factors, and the personality and psychiatric/psychological characteristics of children with dissociative (conversion) disorders is important for early diagnosis and treatment and long-term prognosis.

## Methods

### Participants and procedures

Sixty-six patients with dissociative (conversion) disorders were identified who met the inclusion and exclusion criteria between January 2017 and July 2019 at the Children’s Hospital of Chongqing Medical University, the first pediatric medical center in western China. The inclusion criteria were a diagnosis of dissociative (conversion) disorders according to DSM-5 criteria [1], age 7–18 years, ability to read, and knowledge of the language. The exclusion criteria were the presence of a physical/somatic illness, psychotic disorders, pervasive developmental disorder, or substance abuse/dependence. The study was approved by the ethics committee of the hospital and written informed consent was obtained from the parents of the patients.

During the hospitalization of these patients, clinicians performed careful examinations of them to rule out physical/somatic illnesses. Additional diagnostic procedures were conducted, when necessary, such as computed tomography scans, magnetic resonance imaging, electroencephalography, echocardiography, electrocardiograms, lung-function testing, and gastroscopy. After that, experienced clinical psychologists conducted complete interviews and assessments (including completing the scales/questionnaires) of each patient. When these patients were diagnosed with dissociative (conversion) disorders, all of them were treated with a combination of individual psychotherapy, pharmacotherapy, and family therapy.

When we determined the patients to be included in the study, we created a detailed clinical data and registration form for each patient. Existing clinical data on the patients and their family members, including gender, age at onset, precipitants (stressors), main signs and symptoms, diagnosis, family history, socio-cultural factors, family factors, school factors, the results of the measures described below, and auxiliary examination results were obtained from medical records. The measures were reviewed for incomplete information/missing data, and missing data on disease development, degree of treatment cooperation, and prognosis were obtained via telephone or out-patient follow-up.

### Measures

#### Eysenck personality questionnaire (EPQ) for children

The EPQ was designed to assess personality traits. The Chinese version was revised by Gong et al. [17] according to the original version provided by Eysenck, which contains 88 items that comprise four subscales: Extraversion–introversion (E), with higher scores indicating greater extroversion; Neuroticism (N), with higher scores indicating less stable emotions; Psychoticism (P), with higher scores indicating higher psychoticism; and Lie (L), which is used to measure the tendency of the individual to “conceal.” The original score for each subscale is the sum of the responses (agreement or disagreement) for that subscale. The raw score was converted to a standard score (T-score) based on the domestic norm. If the T-scores of neuroticism were higher than 56.7, the results were classified as unstable emotions; if the T-scores of neuroticism were less than 43.3, the results were classified as stable emotions. If the T-scores of psychoticism were higher than 61.5, the results indicated psychoticism. Moreover, Extraversion–introversion T-scores less than 43.3 and more than 56.7 indicated introversion and extraversion, respectively. The validated Chinese version of the EPQ has adequate reliability and validity among Chinese patients [18, 19].

#### Raven standard progressive matrices (RSPM)

The RSPM, which contains 60 items, was used to evaluate general cognitive ability. The total score is calculated by summing scores of the 60 items, and classified into six levels of intelligence: 1 (≥ 130 = superior), 2 (129–110 = good), 3 (109–90 = average), 4 (89–80 = below average), 5 (79–70 = borderline), and 6 (≤69, deficient). The advantage of the RSPM is that the test’s subject matter is not restricted by culture, race, or language, and the reliability and validity of the RSPM in Chinese children and adolescents have been demonstrated [20].

#### Depression self-rating scale for children (DSRSC)

A Chinese version of the DSRSC was prepared by Su et al. [21], by initially translating the DSRSC into Chinese, after which an individual not directly involved in the study translated their version back into English (back translation). This confirmed that the back translation was nearly identical to the original version. The DSRSC consists of 18 items related to depression in children and adolescents. Evidence suggests that scores ≥15 effectively discriminate between depressed and non-depressed youth. The reliability and validity of the Chinese version of the DSRSC have been confirmed [21].

#### Screen for child anxiety related disorders (SCARED)

The SCARED was used to assess childhood anxiety disorders. The scale was translated into Chinese by Wang et al., and then a professor of English at the Xiangya Medical College translated it back to English, which showed it was faithful to the original text [22]. The scale includes five factors, and evidence suggests that scores ≥23 effectively discriminate between anxious and non-anxious youth. The reliability and validity of the Chinese version of the SCARED have been confirmed [23].

#### Brief psychiatric rating scale (BPRS)

The BPRS, which is used to rate the symptom severity of various classes of psychiatric problems, was used to evaluate clinical efficacy. A reduction in symptom severity ≥75% is considered curative, ≥50% represents significant progress, ≥25% represents progress, and < 25% is considered ineffective [24]. The Chinese version of the BPRS has been validated to be a suitable instrument for the description, measurement, and classification of psychopathology in a Chinese sample [25].

### Statistical analyses

IBM SPSS V. 26.0 was used to conduct the statistical analyses. Categorical variables are presented as frequencies and percentages, whereas continuous variables are presented as means and standard deviation (SD). The independent-samples *t*-test was used to assess between-group differences on the RSPM. Bivariate logistic regression was used to analyze the variables associated with clinical efficacy. Multivariate logistic regression was subsequently used to estimate the adjusted associations between the variables identified in the bivariate analyses and clinical efficacy. A *P* < 0.05 was considered statistically significant; all the tests were two-tailed.

## Results

### Demographic and environmental information

A total of 66 eligible children and adolescents were enrolled in the study. Their demographic and environmental information are summarized in Table 1. Of the 66 patients, 57.6% were male and 42.4% were female and their mean age at onset was 9.97 years. Only four patients had a positive family history, of which two patients had a family history of psychotic disorders, and the other two patients had a family history of dissociative disorders and anxiety disorders. Over two-thirds of the patients (69.7%) had antecedent stressors, the most common of which were scolding or punishment (*n* = 15), learning problems (*n* = 10), school bullying (*n* = 6), changing schools (*n* = 5), and family conflict or parental divorce (*n* = 4). Approximately 3 in 10 patients was an only-child, 45.5% were left-behind adolescents, and roughly one-quarter were from a single-parent family. The majority of the patients had a close relationship with their parents, while the rest did not. Only 15.2% of fathers and of mothers 6.1% had an educational level of junior college or above. Nearly six of 10 also had harmonious relationships with classmates, whereas 40.9% had inharmonious relationships with classmates, with six patients having a history of school bullying. The vast majority of families (80.3%) cooperated with treatment.

**Table 1:** Demographic and environmental information of children with dissociative (conversion) disorders

Characteristics	Total (*N* = 66)
Age of onset (years), mean (SD), range	9.97 (1.98), 7–15
Sex, *n*(%)	
Male	38 (57.6)
Female	28 (42.4)
Family history, *n* (%)	
Positive	4 (6.1)
Negative	62 (93.9)
Inducement, *n* (%)	
Have inducement	46 (69.7)
Have not inducement	20 (30.3)
Place of Residence, *n* (%)	
Rural	38 (57.6)
Urban	28 (42.4)
Left-behind adolescents, *n* (%)	
Yes	30 (45.5)
No	36 (54.5)
Number of friends, *n* (%)	
0	3 (4.6)
1–2	14 (21.2)
3–4	16 (24.2)
≥ 5	33 (50.0)
Only-child, *n* (%)	
Yes	20 (30.3)
No	46 (69.7)
Relationship with parents, *n* (%)	
Close	36 (54.5)
Not close	30 (45.5)
Family monthly income, *n* (%)	
< 2000¥	12 (18.2)
2000–5000¥	19 (28.8)
5000–10,000¥	22 (33.3)
> 10,000¥	13 (19.7)
Parental rearing style, *n* (%)	
Despotic	29 (43.9)
Democratic	32 (48.5)
Permissive	5 (7.6)
Father’s educational level, *n* (%)	
Primary school and below	20 (30.3)
Junior high school to senior high school	36 (54.5)
Junior college or above	10 (15.2)
Mother’s educational level, *n* (%)	
Primary school and below	26 (39.4)
Junior high school to senior high school	36 (54.5)
Junior college or above	4 (6.1)
Single-parent family, *n* (%)	
Yes	16 (24.2)
No	50 (75.8)
Learning achievement, *n* (%)	
Grade A	18 (27.3)
Grade B	21 (31.8)
Grade C	13 (19.7)
Grade D	14 (21.2)
Relationship with classmates, *n* (%)	
Harmonious	39 (59.1)
Inharmonious/School bullying	27 (40.9)/ 6 (9.1)
Degree of cooperation of family members, *n* (%)	
Cooperative	53 (80.3)
Uncooperative	13 (19.7)

### Clinical manifestations

Table 2 shows the clinical manifestations of children with dissociative (conversion) disorders. Conversion disorders (89.4%) was the most common manifestation, with 15 patients having motor symptoms (i.e., paralysis, tremor, dystonic movement, or dysphonia), 16 having attacks or seizures, 32 having sensory symptoms (i.e., anesthesia, hyperesthesia, or a visual or hearing disturbance), and 6 having more than one type of conversion symptom. Dissociative disorders (i.e., dissociative amnesia, dissociative trance, depersonalization, or derealization) were only observed in 14 patients, and 7 patients had mixed dissociative and conversion disorders. These symptoms could occur either at school or at home, and some patients experienced these symptoms both at home and school. Comorbidity with anxiety or depressive disorders was present in 45.5% of the sample. Twenty-five patients (37.9%) had other comorbid somatic symptoms, with headache, abdominal pain, nausea, dizziness, and fatigue being the most common.

**Table 2:** Clinical manifestations of children with dissociative (conversion) disorders (*N* = 66)

Clinical manifestations	*n* (%)
Dissociative disorders	14 (21.2)
Conversion disorders	59 (89.4)
Motor symptoms	15 (22.7)
Attacks/ Seizures	16 (24.2)
Sensory symptoms ^a^	32 (48.5)
≥ two types of conversion disorders	6 (9.1)
Mixed dissociative (conversion) disorders	7 (10.6)
Comorbidity with anxiety or depressive disorders	30 (45.5)
Comorbidity with anxiety disorders	28 (42.4)
Comorbidity with depressive disorders	19 (28.8)
Comorbidity with other somatic symptoms	25 (37.9)

^a^Of these 32 patients, 2 patients had two types of sensory symptoms

After the combined treatment, the BPRS results showed treatment was curative for 62.1% of patients, that 27.3% made significant progress, 9.1% made progress, and treatment was ineffective for one patient. Therefore, we divided the patients into two groups based on the clinical efficacy of treatment: a curative group (*n* = 41) and a non-curative group (*n* = 25). In addition, 21 of the 41 curative patients had comorbid anxiety or depressive disorders, while 9 of the 25 non-curative patients had comorbid anxiety or depressive disorders.

### Assessments of patients

Sixty-four patients agreed to complete the EPQ, the results of which are shown in Table 3. Almost 6 out of 10 patients had an introverted personality, and about half had unstable emotions. Psychoticism was found in only six patients. Table 4 presents the results of the RSPM, which was completed by 54 patients. The mean RSPM score was 101.22, with 25.4% of participants having scores between 129 and 110 (good), 26.9% having scores between 109 and 90 (average), 11.9% having scores between 89 and 80 (below average), 10.4% having scores between 79 and 70 (borderline), 3.0% having scores ≥130 (superior), and two having scores ≤69 (deficient). Ad hoc tests found patients with anxiety or depression had significantly lower RSPM scores than other patients (*P* < 0.01), but no significant difference in RSPM scores was found between patients with and without precipitants.

**Table 3:** EPQ in children with dissociative (conversion) disorders (*N* = 64)

Personality	*n* (%)
Extraversion–Introversion	
Extraversion	9 (14.0)
Introversion	38 (59.4)
Intermedius	17 (26.6)
Neuroticism	
Stable emotions	6 (9.4)
Relatively stable emotions	24 (37.5)
Unstable emotions	34 (53.1)
Psychoticism	
Yes	6 (9.4)
No	58 (90.6)

**Table 4:** RSPM in children with dissociative (conversion) disorders (*N* = 54)

	Total	Precipitant		Anxiety or depression	
		+ (*n* = 39)	- (*n* = 15)	+ (*n* = 26)	- (*n* = 28)
Mean score (SD)	101.22 (19.93)	101.59 (18.44)	100.27 (24.06)	91.88 (18.67)	109.89 (17.18)
*t*		0.22		3.68^**^	

^**^P < 0.01

### Logistic regression for variables predicting prognosis

Bivariate logistic regression was conducted to determine which variables predicted clinical efficacy. The outcomes were coded as 0 and 1, where 0 represents the curative group, and 1 represents non-curative group. The independent variables included demographic variables (age of onset, sex, family history, and precipitant), environmental variables (place of residence, being a left-behind adolescent, number of friends, being an only-child, relationship with parents, family monthly income, parental rearing style, parents’ educational level, single-parent family, learning achievement, relationship with classmates, degree of cooperation of family members), comorbid anxiety or depressive disorders, and comorbidity with other somatic symptoms. The variables that were found to be significant in the bivariate logistic regression were relationship with parents, father’s educational level, and degree of cooperation of family members. However, it was not clear whether these variables made an independent contribution to clinical efficacy or acted in combination with other variables. Thus, we analyzed these three variables simultaneously with multivariate logistic regression. These results of the bivariate and multivariate regression analyses are presented in Table 5.

**Table 5:** Bivariate and multivariate logistic regression of factors associated with clinical efficacy (curative and non-curative)

Factors	Bivariate			Multivariate		
	OR	95%CI	*P* value	OR	95%CI	*P* value
Relationship with parents						
Not close	Reference					
Close	0.218	0.075–0.635	0.005^**^	0.133	0.034–0.517	0.004^**^
Father’s educational level						
Junior college or above	Reference					
Junior to senior high school	0.242	0.053–1.101	0.066	0.099	0.016–0.629	0.014^*^
Primary school and below	0.143	0.026–0.774	0.024^*^	0.076	0.010–0.556	0.011^*^
Degree of cooperation of family members						
Uncooperative	Reference					
Cooperative	0.192	0.052–0.716	0.014^*^	0.158	0.034–0.724	0.018^*^

^*^P < 0.05, ^**^P < 0.01

The multivariate analysis revealed that having a close relationship with parents (OR = 0.133), a father’s educational level of junior to senior high school (OR = 0.099) and primary school and below (OR = 0.076), and a family that cooperated with the treatment (OR = 0.158) were associated with a lower risk for a non-curative outcome. The multivariate regression model was found to have a good fit according to the Hosmer and Lemeshow goodness of fit test (*P* = 0.489).

## Discussion

Dissociative (conversion) disorders are more common in developing countries. Although there currently are no solid data on the prevalence of dissociative (conversion) disorders in children and adolescents in China, several studies have investigated the prevalence rates of dissociative disorders in both clinical and nonclinical populations in the Chinese context. One study found that 4.52% of 177 college students in Hong Kong may have a DSM-5 dissociative disorder [26]. Another study, which investigated the prevalence rates of dissociative disorders in psychiatric inpatients in Taiwan, reported that 19.5% of psychiatric inpatients were diagnosed as having a dissociative disorder [9]. Dissociative (conversion) disorders are usually related to psychological and environmental factors. Antecedent stressors are identified in the large majority of children, including social or family stress, adverse life events, and traumas, such as violence and abuse. Previous studies have shown that pathological dissociation is associated with psychological trauma, especially childhood trauma [27, 28]. Furthermore, the dissociation of psychological functions after trauma may even separate the mental representations of the body from emotional awareness, causing alexithymia and several somatoform symptoms [29, 30]. However, not all traumatized children develop dissociative symptoms. Other factors, such as family environment, affect regulation ability, and attachment may also affect the relationship between dissociation and trauma [31, 32]. Barach first proposed a relationship between attachment theory and dissociation [33]. Disorganized attachment has been proposed as a mediating mechanism in the relationship between childhood trauma and dissociation, which may be even more central to the development of dissociation than trauma itself [34, 35]. Children are particularly susceptible to the above triggering factors due to their immature personality and increased sensitivity to adverse situations, and they easily react in this way to mental stress [4, 12]. Our research found that about two-thirds of the patients had antecedent stressors, and that the most common stressors were similar to those reported in previous studies, mainly school and family factors [6, 7]. This is reflected in the fact that about two-fifths of patients had inharmonious relationships with classmates, of which, six patients had a history of school bullying, nearly a quarter of the patients were from a single-parent family, and 40.9% of the patients had a school grade below B. In addition, nearly half of our patients being left-behind children and their migrating parents have limited communication with their children, often discussing only their children’s learning situations rather than their emotional state or peer interaction. These disadvantages increase their children’s anxiety about learning and have a negative effect the left-behind child’s emotional development, self-awareness, mental health, and social behaviors [2, 36].

Motor symptoms and non-epileptic seizures are the most common symptoms in children with dissociative disorders, and multiple conversion symptoms are the norm [4, 14]. Conversion symptoms accounted for the majority symptoms in our study and only one-fifth of the patients presented with dissociative symptoms. Yet, we found nearly half of the patients had sensory symptoms, which may be due to the fact that, compared to sensory symptoms, motor symptoms are more easily identified by auxiliary examination, and it is not easy for lower level hospitals to diagnose patients with sensory symptoms. Hence, patients with sensory symptoms may be relatively more common in our hospital because it is a tertiary hospital. Moreover, previous studies have found that some patients have mixed dissociative and conversion disorders, and many children may present with concomitant, nonspecific somatic complaints, as found in our own research [14, 37]. Anxiety and depression are frequently found in patients with dissociative (conversion) disorders (45.5% of the patients in the present study had anxiety or depressive disorders comorbidity), and they can increase disease severity in children [16]. Thus far, no study has clarified the relationship between depression or anxiety and dissociative (conversion) disorders. Hence, it remains unclear whether dissociative (conversion) disorders are a consequence of persistent depression or anxiety symptoms, or vice versa.

The comorbidity of dissociative (conversion) disorders with borderline personality disorder is well documented. Borderline personality disorder is characterized as a pattern of instability in interpersonal relationships and emotional regulation, with psychotic-like symptoms and marked impulsivity [37]. We used the EPQ, which was designed to assess personality traits, in our study, and found about half of the patients had unstable emotions and a small percentage of the patients exhibited psychoticism. Studies of neurocognitive functioning in patients with dissociative (conversion) disorders have a found decrease in intelligence quotient (IQ) and neurocognitive function at baseline [13]. It has been hypothesized that the decreased cognitive functioning of patients with dissociative (conversion) disorders may be due to upregulation or priming of the stress system in various ways [38]. In addition, comorbid anxiety or depression may affect the cognitive functioning of these patients [39]. However, other studies suggest that children with dissociation have poor cognitive ability, which results in ineffective coping with stress, and that lower IQ scores were significantly associated with the poor adjustment in school, which induces dissociative (conversion) disorders [40]. The RSPM results in our study showed that the mean score of patients was 101.22, which was classified as average intelligence; only 13.4% of the patients had a level of intelligence that was borderline or deficient. This means that our patients’ average level cognitive function had not decreased significantly. In addition, we found that the patients with comorbid anxiety or depression had significantly lower scores on the RSPM than patients without anxiety or depression, and this is consistent with previous research. However, no significant difference in RSPM scores was found between patients with and without stressors.

A multidisciplinary approach is thought to be the most efficacious treatment for dissociative (conversion) disorders. A comprehensive psychotherapeutic approach is the basis of the treatment, which includes cognitive-behavioral therapy, psychodynamic therapy, and hypnosis. Other treatment approaches, which include behavioral training, family therapy, and medication in cases with concomitant disorders, produce the best therapeutic effects [12]. The first treatment approach is to stabilize the child’s emotions by building a therapeutic alliance based on trust and hope, using specific therapeutic relaxation techniques and ensuring the child can express his/her emotions and needs. For patients with previous trauma, family therapy can improve family communication by correcting interaction patterns that promote dissociation, and help parents deal with guilt or denial related to previous trauma. Family work to promote attachment experiences are also important [4]. Patients who suffer from comorbid anxiety-depressive disorders need to be treated with antidepressants and anxiolytics [7, 12]. In addition, cognitive-behavioral therapy is an effective intervention for patients with cognitive impairments, which can change the outcome of dissociative (conversion) disorders [40, 41].

Most children recover completely within a few weeks after therapeutic interventions. However, in rare cases, the symptoms can last much longer, sometimes several months or even years [2, 42]. To explore factors related to prognosis, we conducted logistic regression to analyze the factors that influence clinical efficacy. The results revealed that the children’s relationship with their parents, their father’s level of education, and the degree of cooperation of family members were significantly associated with prognosis. Family therapy is an important part of the treatment of dissociative (conversion) disorders, which requires improving the relationship between children and their parents, enabling children to express their feelings, and helping their parents to deal with their child’s emotional situation and needs [4]. Therefore, promoting the relationship between parents and children is helpful for improving the prognosis. Barriers to treatment include difficulty communicating the diagnosis to patients and their family members, and it is not an unusual response for parents to have doubts about the diagnosis of dissociative (conversion) disorders. Many parents find it difficult to understand that emotional factors and mental states can lead to obvious physical symptoms, which indicate a serious physical illness [12]. So, nearly all patient’s parents have some initial resistance or disbelief about the diagnosis, and lack of understanding, acceptance, or both, which results in poor cooperation with the therapeutic process and postpones recovery [42]. In that case, it is important to address parents’ questions and potentially negative reactions effectively, to explain that the child’s symptoms are not fake or intentionally driven by the child, and to encourage parents to cooperate with the treatment plan [7]. Interestingly, we found that the higher the father’s educational level, the worse the patient’s prognosis was. The reason for this may be that fathers with more education may be more assertive, so it is easier for them to question the diagnosis of a dissociative (conversion) disorder, and most Chinese families are dominated by fathers, resulting in treatment incompatibility. At the same time, lower self-esteem, fear, and exaggerated ambitions have been observed in these patients, leading to increased difficulty with treatment [12]. However, we should be skeptical about this result, as this may be due to a special situation in specific cases that cannot be generalized, or it may be caused by the small sample size and it needs further research to verify it.

This study has several limitations. First, the sample size of this study is not large and we only recruited clinical samples in our center; thus, selection bias may exist in our study and we cannot guarantee that the sample is representative. Hence, future multi-center studies with large samples are necessary. Second, there is no validated Chinese version of structured diagnostic interviews for children. Therefore, a revision of validated structured interviews would be helpful for future research. Third, there may have been unrecognized problems with the translation of the measures. However, these problems are unlikely to be serious because the Chinese version of these measures have been shown to have adequate reliability and validity, and the results were interpreted by Chinese psychologists. Fourth, although the EPQ is widely used in China and it has been found to have high reliability and validity, it has not been revised for a long time and it is not consistent with the current dimensional approach to the study of personality in children (i.e., emotional stability, extraversion, imagination, benevolence, and conscientiousness). Therefore, personality assessments may need to be improved in the future. Fifth, a comparison between patients with dissociative (conversion) disorders and patients in other clinical populations would have been valuable to verify our findings. Regardless, of these limitations, the present study has significant implications because most pediatricians do not understand dissociative (conversion) disorders, and we have described the clinical characteristics, socio-cultural and environmental factors, and psychiatric/psychological characteristics of children with dissociative (conversion) disorders.

This should increase clinicians’ knowledge about these disorders, and help them avoid misdiagnosis. In addition, we have explored the factors that affect prognosis, which entail improving the relationship and increasing the communication between children and their parents, and striving for trust and cooperation with parents, all of which will help improve the prognosis of these disorders.

## Conclusions

This study describes the clinical characteristics, socio-cultural and environmental factors, and psychiatric/psychological characteristics of children with dissociative (conversion) disorders in western China. We also explored the factors related to prognosis, which is useful for clinical treatment and predicting 0prognosis. Prospective longitudinal studies at multiple centers with larger sample sizes are needed to verify our findings.

## Data Availability

The datasets used during the current study are available from the corresponding author upon reasonable request.

## Abbreviations

BPRS: Brief Psychiatric Rating Scale
DSM-5: Mental Disorders - Fifth edition
DSRSC: Depression Self-Rating Scale for Children
EPQ: Eysenck Personality Questionnaire
IQ: Intelligence quotient
OR: Odds ratio
RSPM: Raven Standard Progressive Matrices
SCARED: Screen for Child Anxiety Related Disorders
SD: Standard deviation
